# Molecular tools for studying the major malaria vector *Anopheles funestus*: improving the utility of the genome using a comparative poly(A) and Ribo-Zero RNAseq analysis

**DOI:** 10.1186/s12864-015-2114-z

**Published:** 2015-11-14

**Authors:** Gareth D. Weedall, Helen Irving, Margaret A. Hughes, Charles S. Wondji

**Affiliations:** Vector Biology Department, Liverpool School of Tropical Medicine, Pembroke Place, Liverpool, L3 5QA UK; Centre for Genomic Research, Institute of Integrative Biology, University of Liverpool, Crown Street, Liverpool, L69 7ZB UK

**Keywords:** *Anopheles funestus*, Mosquitoes, Transcriptomics, RNAseq, Genome annotation

## Abstract

**Background:**

Next-generation sequencing (NGS) offers great opportunities for studying the biology of insect vectors of disease. Prerequisites for successful analyses include high quality annotated genome assemblies and that techniques designed for use with model organisms be tested and optimised for use with these insects. We aimed to test and improve genomic tools for studying the major malaria vector *Anopheles funestus*.

**Results:**

To guide future RNAseq transcriptomic studies of *An. funestus*, we compared two methods for enrichment of non-ribosomal RNA for analysis: enrichment of polyadenylated RNA and ribosomal RNA depletion using a kit designed to deplete human/rat/mouse rRNA. We found large differences between the two methods in the resulting transcriptomes, some of which is due to differential representation of polyadenylated and non-polyadenylated transcripts. We used the RNAseq data for validation and targeted manual editing of the draft *An. funestus* genome annotation, validating 62 % of annotated introns, manually improving the annotation of seven gene families involved in the detoxification of xenobiotics and integrated two published transcriptomic datasets with the recently published genome assembly.

**Conclusions:**

The mRNA enrichment method makes a substantial, replicable difference to the transcriptome composition, at least partly due to the representation of non-polyadenylated transcripts in the final transcriptome. Therefore, great care should be taken in comparing gene expression data among studies. Ribosomal RNA depletion of total RNA using a kit designed to deplete human/rat/mouse rRNA works in mosquitoes and, we argue, results in a truer representation of the transcriptome than poly(A) selection. The *An. funestus* genome annotation can be considerably improved with the help of these new RNAseq data and further guided manual gene editing efforts will be of great benefit to the *Anopheles* research community for studies of this insect’s genome and transcriptome.

**Electronic supplementary material:**

The online version of this article (doi:10.1186/s12864-015-2114-z) contains supplementary material, which is available to authorized users.

## Background

*Anopheles funestus* is a major vector species of malaria in sub-Saharan Africa [1]. Similar to the more extensively studied vector species *Anopheles gambiae*, resistance to commonly used insecticides is a growing problem in *An. funestus* [2–9]. Efforts to improve the design and implementation of resistance management strategies require a good understanding of the molecular basis of resistance. Use of genomic tools is paramount to achieve this goal. In contrast to *An. gambiae*, for which significant progress has been made in its genomics since its genome was sequenced more than a decade ago [10], *An. funestus* has received much less attention. However, progress is being made as the genome of *Anopheles funestus* was recently sequenced as part of a program to sequence a number of Anopheles species genomes [11]. The draft genome assembly, in the form of unplaced scaffolds not assembled into chromosome sequences, is publicly available via the VectorBase web resource [12, 13]. The draft genome assembly has been annotated, using the genome annotation pipeline MAKER [14, 15]. However, this annotation has not been extensively manually curated and is inaccurate for a number of known genes of interest such as cytochrome P450 monooxygenase and glutathione S-trasferase (GST) genes. Thus, more efforts are currently needed to maximise the usefulness of the *An. funestus* genome and allow more studies to be performed such as transcriptome profiling or genome-wide association studies. One approach to optimise the quality of this genome is to use RNAseq to improve genome annotation.

RNAseq is a powerful method to study the transcriptomes of organisms, providing a rich dataset allowing transcriptional profiling as well as the identification of novel transcripts, alternative splicing and detection of expressed sequence polymorphisms [16, 17]. However in the case of Anopheles mosquitoes, limited information have been generated to determine the best methodology for library preparation to ensure an accurate profiling of transcriptomes, notably between the enrichment of polyadenylated (poly(A)) RNA transcripts and the alternative method of ribosomal RNA (rRNA) depletion. Methods of mRNA enrichment have been shown to have an effect on the observed transcriptome [18–21]. Comparisons of poly(A) mRNA enrichment with rRNA depletion methods report that the depletion methods result in more reads aligned outside of annotated gene coding regions [18, 20]. This may be due to the presence of novel transcripts and/or a greater proportion of genomic DNA in the library. Also, the greater number of reads aligned within introns in depletion libraries could be derived from immature transcripts [21]. Depletion libraries show more even read distribution less 3′ bias than poly-A libraries [18].

In malaria-transmitting mosquitoes, insecticide resistance is often due to ‘metabolic resistance’: up-regulation of genes involved in detoxification, such as cytochrome P450 and GST genes. This is particularly the case in *An. funestus*, for which pyrethroid resistance is caused by metabolic resistance and not by target site resistance, such as mutations in the voltage gated sodium channel gene. As there are no known genomic biomarkers to detect metabolic resistance, transcriptomic analyses are necessary. These analyses have commonly used microarrays to detect up-regulated genes [4–6, 8]. RNAseq may offer advantages over microarrays in that unannotated, or poorly annotated, transcripts can be defined and corrected using the sequence alignment, which is more data-rich than the results of microarray analysis. This data richness includes information on alternative splicing, as well as non-protein-coding RNA transcripts (ncRNA) that may play a role in differential gene expression.

Here, we perform an RNAseq experiment on *Anopheles funestus* (FANG strain), primarily to compare two methods to enrich messenger RNA (mRNA) relative to ribosomal RNA (rRNA): ribosomal depletion and poly(A) selection. We compare the results of both methods applied to total RNA from the same individual mosquitoes and identify the most differentially represented transcripts between the methods to identify non-polyadenylated transcripts. We use the RNAseq data along with published *de novo* assemblies of *Anopheles funestus* transcripts [22, 23] to improve the genome annotation for selected genes, focusing on detoxification associated gene families.

## Results and discussion

### Preparation of transcriptome sequencing libraries

Total RNA was extracted from pools of whole mosquitoes of the *An. funestus* FANG colony. Extracts showed a pink colouration, probably due to eye pigments. It was not known if this would affect the sequencing library quality, so one sample was split and bead clean-up carried out on one of the sub-samples, to compare results with its uncleaned counterpart and assess whether this discolouration had any effect on the sequencing results. BioAnalyzer traces of total RNA (Additional file 1) showed evidence of the ‘hidden break’ reported in other insect total RNA samples [24]: two peaks of approximately 2000 bp representing 18S rRNA and the 28S rRNA subunits alpha and beta. The total RNA traces also showed some ‘spikiness’ the cause of which was unclear. The profile of degraded RNA tends to look smoother, with a broad peak extending left of the major 18S/28S rRNA peaks. Whether this spikiness would affect the sequencing results was not known at this stage.

After Ribo-Zero rRNA depletion or poly(A) mRNA enrichment, BioAnalyzer traces suggested that all samples were of sufficient quality for RNAseq library preparation, with no obvious quality differences among samples within each method (Additional file 1). The amount of rRNA remaining in the samples appeared to be much lower for Ribo-Zero depleted samples than for poly(A) enriched samples, where even after 3 rounds of poly(A) selection, samples displayed a large peak near 2000 bp (Additional file 1). Later analysis indicated that this peak may represent mitochondrial 16S rRNA.

Initial quality checking of the sequence read data showed that the 8 libraries were evenly represented in the sequenced pool (median 61,229,870 reads per library; range 44,384,316 to 68,640,804 reads) and of good quality, with little read trimming due to sequenced adapters or low quality base calls (full details in Additional file 1).

### Alignment of sequence reads to reference genome

All read libraries were aligned to a reference consisting of 1392 scaffolds derived from the *Anopheles funestus* FUMOZ colony with the *An. funestus* mitochondrial genome sequence added. Broad alignment metrics are shown in Table 1. Overall, a median of 35,309,124 (range 21,002,025 to 38,878,773), or 57 % (range 35 % to 59 %) of the reads to be aligned could be aligned. Alignment of spliced reads is more difficult than for unspliced reads and leads to lower proportions of the starting libraries being aligned, but this alone is unlikely to account for the relatively low alignment rate. The incompleteness of the reference genome sequence and high levels of sequence polymorphism and genetic divergence between laboratory colonies FANG (derived from Angola) and FUMOZ (from Mozambique, used for sequencing the reference genome) may also contribute to it. A greater proportion of the poly(A) than the Ribo-Zero libraries tended to be aligned (mean difference 11 %, median 8 %). For the sample where an initial bead clean-up was applied (“F1_XP”), the proportion of reads aligned for the Ribo-Zero library (“F1_XP_RZ”; 55 %) was greater than for the uncleaned sample (“F1_RZ”; 35 %). This difference was not seen for poly(A) libraries (59 % for both “F1_XP_PA” and “F1_PA”). This suggests that the purity of the sample may affect the efficacy of the rRNA depletion reaction, which is supported by the ribosomal RNA mapping results shown in Table 2.

**Table 1:** Metrics describing the transcriptome alignments

Sample ID	Reads to align (R1 + R2)	Aligned reads (%)^a^	Aligned R1 (%)^b^	Aligned R2 (%)^b^	Aligned in pair (%)^b^	Properly paired (%)^b,c^	Singleton (%)^b^
F1_XP_RZ	67,895,442	37,480,868 (55 %)	20,409,963 (54 %)	17,070,905 (46 %)	28,766,542 (77 %)	26,655,176 (71 %)	8,714,326 (23 %)
F1_XP_PA	65,605,318	38,878,773 (59 %)	21,731,643 (56 %)	17,147,130 (44 %)	29,122,168 (75 %)	27,271,540 (70 %)	9,756,605 (25 %)
F1_RZ	60,479,632	21,002,025 (35 %)	11,311,755 (54 %)	9,690,270 (46 %)	15,677,600 (75 %)	14,192,682 (68 %)	5,324,425 (25 %)
F1_PA	63,555,450	37,671,216 (59 %)	20,881,499 (55 %)	16,789,717 (45 %)	28,690,598 (76 %)	26,499,330 (70 %)	8,980,618 (24 %)
F2_RZ	43,733,908	24,067,157 (55 %)	13,121,886 (55 %)	10,945,271 (45 %)	18,560,162 (77 %)	17,136,240 (71 %)	5,506,995 (23 %)
F2_PA	60,656,692	35,610,912 (59 %)	19,661,565 (55 %)	15,949,347 (45 %)	27,281,408 (77 %)	25,371,888 (71 %)	8,329,504 (23 %)
F3_RZ	54,693,906	25,673,135 (47 %)	13,831,002 (54 %)	11,842,133 (46 %)	19,086,154 (74 %)	17,431,254 (68 %)	6,586,981 (26 %)
F3_PA	60,211,066	35,007,336 (58 %)	19,424,414 (55 %)	15,582,922 (45 %)	26,507,306 (76 %)	24,701,646 (71 %)	8,500,030 (24 %)

^a^% of reads to align

^b^% of aligned reads

^c^Properly paired means both read and its mate are mapped to opposing strands of the reference sequence, with 3′ ends innermost and within the allowed distance from each other (mean 0 bp, standard deviation 100 bp)

**Table 2:** Tag counts for ten putative rRNA genes

GeneID	Description	Tag count, F1_XP_RZ	Tag count, F1_XP_PA	Tag count, F1_RZ	Tag count, F1_PA	Tag count, F2_RZ	Tag count, F2_PA	Tag count, F3_RZ	Tag count, F3_PA
AFUN015486	28S	20,426	10,311	120,345	16,664	19,706	28,057	95,593	20,651
AFUN015548	28S	47,771	21,402	154,805	31,516	48,525	54,305	145,807	41,643
AFUN015372	18S	3,064	4,289	24,027	4,787	6,759	8,400	17,239	6,504
AFUN015629	5.8S	0	0	0	0	1	0	0	0
AFUN015410	5.8S	13	6	6	1	0	3	4	5
AFUN015687	5.8S	0	0	0	0	0	0	0	0
AFUN015706	5.8S	0	0	0	0	0	0	0	0
AFUN015471	5.8S	1	0	0	0	0	0	0	0
16S-rRNA	16S (mito.)	768,823	2,673,376	402,555	2,351,745	272,148	1,927,215	491,163	2,382,882
12S-rRNA	12S (mito.)	51,260	6,613	20,543	7,110	10,995	3,066	21,513	3,032
Total (%)^a^		891,358 (3.86 %)	2,715,997 (11.17 %)	722,281 (5.49 %)	2,411,823 (10.34 %)	358,134 (2.42 %)	2,021,046 (9.20 %)	771,319 (4.78 %)	2,454,717 (11.28 %)

^a^Total rRNA gene tag counts as a % of total aligned tags for each sample

### Tag counting for annotated genomic features and analysis of the ribosomal RNA content of libraries

Tag counting means quantifying read pairs or singletons (representatives of a single fragment of transcript) aligned in genomic regions annotated as genome features (e.g. protein coding genes or ncRNAs). As the RNAseq protocol used is strand-specific, tags can be aligned sense or antisense to a feature, which mean different things: i.e. that antisense tags come from an antisense transcript, rather than from the primary annotated transcript. Sense and antisense tag counting was performed for each sequence alignment.

To assess how much ribosomal RNA was represented in each sequence library (i.e. the success of the mRNA enrichment procedure) gene features annotated as rRNAs were extracted from the genome annotation. There were ten of these features (Table 2): 8 resulting from automated annotation of the genome assembly and 2 annotated on the mitochondrial genome. For putative nuclear 28S and 18S rRNA genes, poly(A) mRNA enrichment almost always (except for the F1_XP sample pair) resulted in fewer tags from these genes than Ribo-Zero rRNA depletion. Putative 5.8S rRNA genes rarely showed any tags (possibly due to ambiguous read alignment arising from non-uniqueness among multiple 5.8S rRNA copies in the reference genome or to their small size). The mitochondrial 12S rRNA gene showed the same trend as for nuclear 28S and 18S rRNA genes. However, the mitochondrial 16S rRNA gene showed the opposite trend, with more tags in the poly(A) mRNA enriched libraries than in the Ribo-Zero depleted libraries.

**Fig. 1:**
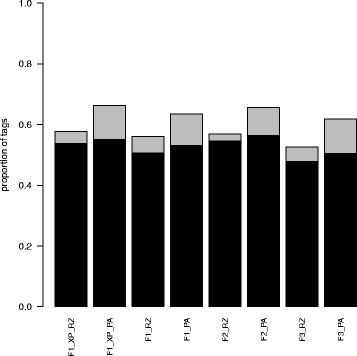
Sense tag counts per annotated gene feature as a proportion of total aligned tags. Grey bars show total sense tag counts as a proportion of total aligned tags while black bars show this after excluding 10 putative rRNA genes

Tag counting is summarised in Table 3. As counting used annotated genomic features, antisense counts should be much lower than sense counts. This was the case, with a ten-fold greater assignment of tags to features in the sense orientation than antisense. The count data generated for each genomic feature is the data analysed in differential gene expression (DGE) analysis. Therefore, in general, the more sense tag-count data available, the greater the power of DGE analysis to detect differential gene expression. Overall, the poly(A) mRNA-enriched libraries produced more “on target” sense tags, as a proportion of all aligned tags (between 7.5 and 9.2 % more than Ribo-Zero), as might be expected given enrichment on the poly(A) tails of mRNA transcripts (Table 3 and Fig. 1). However, after excluding tag counts from 10 putative rRNA genes, the differences between Ribo-Zero and poly(A) samples were less marked and for all samples (between 1.3 and 2.7 % more on-target tags for poly(A) ). Overall, “on target” sense tag counts (excluding rRNA genes) comprised around 47.9 to 56.4 % of the total aligned tags. This relatively low proportion probably reflects the fact that 5′ and 3′ un-translated regions (UTRs) are rarely predicted by automated annotation pipelines, so a large proportion of tags from mRNA transcripts are not counted as such when using gene models mostly comprising protein coding regions. Overall (excluding rRNA genes), our differential “gene expression” analysis would be based on between 6.6 and 13.4 million tags per sample.

**Table 3:** Summary of sense tag counting

Sample	Total tags^a^	Assigned to features, sense orientation (%)^b^	Assigned to non-rRNA features, sense orientation (%)^b,c^	Assigned to features, antisense orientation (%)^b^	Assigned to non-rRNA features, antisense orientation (%)^b,c^
F1_XP_RZ	23,097,597	13,325,951 (57.69 %)	12,434,593 (53.84 %)	1,293,286 (5.60 %)	1,286,090 (5.57 %)
F1_XP_PA	24,317,689	16,120,214 (66.29 %)	13,404,217 (55.12 %)	1,234,142 (5.08 %)	1,216,317 (5.00 %)
F1_RZ	13,163,225	7,379,904 (56.06 %)	6,657,623 (50.58 %)	836,937 (6.36 %)	814,750 (6.19 %)
F1_PA	23,325,917	14,816,364 (63.52 %)	12,404,541 (53.18 %)	1,355,508 (5.81 %)	1,320,535 (5.66 %)
F2_RZ	14,787,076	8,421,844 (56.95 %)	8,063,710 (54.53 %)	1,006,847 (6.81 %)	1,001,013 (6.77 %)
F2_PA	21,970,208	14,411,931 (65.60 %)	12,390,885 (56.40 %)	1,237,662 (5.63 %)	1,218,099 (5.54 %)
F3_RZ	16,130,058	8,495,428 (52.67 %)	7,724,109 (47.89 %)	997,369 (6.18 %)	984,961 (6.11 %)
F3_PA	21,753,683	13,447,922 (61.82 %)	10,993,205 (50.53 %)	1,126,921 (5.18 %)	1,112,413 (5.11 %)

^a^A tag is a read pair or a single, un-paired read

^b^% of “Total tags”

^c^Tags assigned to features after subtracting tags assigned to 10 putative rRNA features

### Comparison of poly(A) mRNA-enriched and Ribo-Zero rRNA-depleted samples

Usually, the variation between samples within a sample group (within-group variation) is smaller than that between samples from different sample groups (overall variation) because the former consists of technical and biological variation only, while the latter also contains variation due to the treatment effect. When the treatment effect is the dominant term of variation, the sample groups can be clearly separated by statistical tools. If the treatment effect is weak compared to the technical and biological variation the samples will be difficult to discriminate based on the data. Here, the treatment effect is the difference between poly(A) and Ribo-Zero mRNA enrichment methods and a large treatment effect indicates that the mRNA enrichment/rRNA depletion method greatly affects the composition of the transcriptome. We assessed these effects.

Initial visual inspection of sequence alignments indicated that the Ribo-Zero libraries showed more ‘background’ alignments (reads aligned in intergenic and intronic regions) than the poly(A) datasets. It is not certain whether this represents real, un-polyadenylated transcripts or possible erroneous sequencing of DNA. However, as samples came from the same pool and were DNase treated prior to being split, any difference in the amount of DNA being sequenced should be due to specific differences in the enrichment/depletion process.

As an initial gene-wise analysis of gene expression levels, reads or read pairs (“tags”) mapped in the sense orientation to annotated gene models were counted for each sample. One caveat to this analysis is that the gene models used to generate tag counts were from a draft genome annotation and therefore were likely to contain errors. 257 of the 13,793 annotated features had sense tag counts of zero in all eight samples and were not analysed. To assess variation in the data, log_2_ sense tag counts per gene were plotted among sample pairs (Additional file 1). These give an indication of how similar are samples belonging to the same group and how different are samples from different sample groups. The associated correlation coefficients are displayed as a correlation heatmap (Additional file 1). Principal component analysis (PCA), using log_2_ sense tag counts per gene, was used to plot each sample relative to all others (Additional file 1). These analyses showed very low technical variation between the cleaned and uncleaned FANG-1 samples and greater biological variation among FANG-1, FANG-2 and FANG-3 samples. Ribo-Zero samples showed more biological variation than poly(A) samples, though this was mainly due to the FANG-2 Ribo-Zero sample, which was a clear outlier (Additional file 1). FANG-2 Ribo-Zero was the smallest sample library, with around 44 million reads compared to around 55–62 million reads for the other samples, which may be associated with this result. Together, these assessments indicate that the mRNA enrichment method has a large effect on the sequenced transcriptomes of the samples.

### Analysis of differential transcriptome composition between mRNA enrichment methods

**Fig. 2:**
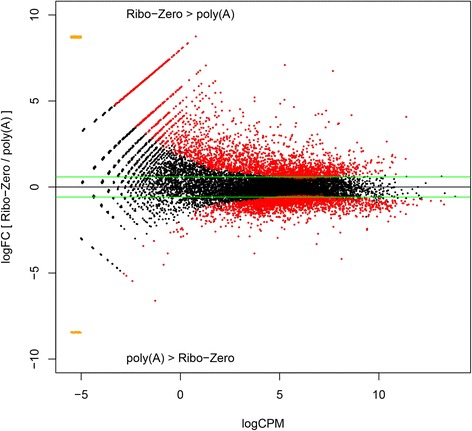
Plot of differential transcript abundance in Ribo-Zero and poly(A) transcriptomes. The y-axis shows log_2_ fold-change of transcript abundance between Ribo-Zero and poly(A) samples. Greater than 0 indicates that a transcript is more abundant in Ribo-Zero samples than in poly(A) samples and *vice versa*, as indicated on the plot. Green lines indicate 1.5-fold change. Red points are significantly differentially represented among treatments (FDR-adjusted *P*-values < 0.05). Orange points are transcripts represented in only one sample, so fold-change could not be calculated. The x-axis shows the log_2_ average abundance of the transcripts in both Ribo-Zero and poly(A) transcriptomes. The shape of the plot and distribution of significantly differentially represented transcripts indicates that low abundance transcripts show more variation among Ribo-Zero and poly(A) transcriptomes due to sampling effects than do higher abundance transcripts

To assess differences between the transcriptomes due to the mRNA enrichment method applied, differential gene expression (DGE) analysis was carried out to identify significantly differentially represented annotated genome features. First, as technical variation due to pre-enrichment sample cleaning of FANG-1 was low and to make full use of the data, the mean tag counts for cleaned and uncleaned samples were calculated and used in the DGE analysis. The data were modelled using a generalized linear model in a one-factor (mRNA enrichment method) experiment with two levels (“Ribo-Zero” and “poly(A)”). Full results of the analysis are shown in Additional file 2. Figure 2 shows the relationship between log_2_ fold change in expression between Ribo-Zero and poly(A) and mean expression level across all samples. The power in the data to discriminate between Ribo-Zero and poly(A) samples (clearly shown by PCA) is reflected in the large number of significantly differentially represented transcripts among the two transcriptomes. This analysis indicates that results based on Ribo-Zero and poly(A) mRNA enrichment may be very different from one another. Great care must therefore be taken when comparing results derived using the different methods.

In order to assess why the composition of Ribo-Zero and poly(A) transcriptomes differ so much, we looked at the 20 most differentially represented genome features in each group (ordered by FDR-adjusted *P*-value). Table 4 lists the 20 features most over- or under-represented in the Ribo-Zero transcriptome relative to poly(A). The two most over-represented features in the Ribo-Zero transcriptomes were ncRNAs, suggesting a large population of non-polyadenylated transcripts, including many ncRNAs, may exist in the cells and be under-represented in poly(A) transcriptomes. Two histone genes (histone H2A and H3) were over-represented in the Ribo-Zero transcriptome, consistent with the occurrence of replication-dependent, unpolyadenylated and replication-independent, polyadenylated histone forms [19, 25]. Also, three genes with Cadherin domains were over-represented in the Ribo-Zero transcriptome. In other systems, Cadherin is among a set of genes controlled at the translational level by cytoplasmic polyadenylation [26]. If this were occurring in *An. funestus*, we would expect transcript forms with short poly(A) tails to be over-represented in the Ribo-Zero transcriptome. Further investigation would be needed to confirm if this is the case. Among the genes over-represented in the poly(A) transcriptome were a number of putative core metabolic genes such as transcription-associated proteins and ribosomal proteins. A study of poly(A) tail length in yeast showed that ribosomal proteins had long poly(A) tails [27]. We speculate that preferential enrichment of transcripts with longer poly(A) tails may affect the resulting transcriptome composition.

**Table 4:** The most significantly differentially represented genome features between Ribo-Zero and poly(A) transcriptomes

Gene ID	logFC (Ribo-Zero/poly(A))^a^	logCPM^b^	Adjusted *P*-value^c^	Description
AFUN015353	6.73	7.69	1.31E-89	Signal recognition particle (ncRNA)
AFUN015615	7.08	5.27	2.65E-75	Signal recognition particle (ncRNA)
AFUN010049	5.05	5.25	3.80E-71	Histone H3
AFUN015157	4.18	6.81	5.86E-62	-
AFUN009381	4.64	5.73	7.90E-58	-
AFUN008241	4.17	8.62	2.49E-47	-
AFUN003688	4.43	7.35	1.91E-46	-
AFUN014500	3.78	7.48	7.14E-44	-
AFUN014369	3.71	5.84	1.51E-41	-
AFUN015010	4.84	5.04	9.84E-41	-
AFUN002493	3.46	7.32	3.88E-40	Unknown, contains cadherin domains
AFUN000145	3.49	4.93	2.31E-39	-
AFUN008247	4.98	3.75	4.38E-37	-
AFUN005891	5.19	3.75	7.85E-37	-
AFUN011958	4.05	4.83	2.41E-36	Retrotransposon, putative
AFUN011227	3.50	5.80	1.43E-35	Unknown, contains cadherin domains
AFUN008652	3.03	5.65	3.24E-34	Histone H2A
AFUN011228	3.62	5.06	3.96E-33	Unknown, contains cadherin domains
AFUN000984	2.81	6.53	2.55E-32	Unknown, contains PH-domain
AFUN002265	2.94	5.61	4.67E-32	odz/ten-m gene, putative
AFUN010223	−2.72	6.94	5.66E-26	60S ribosomal protein L39
AFUN004938	−2.45	5.25	4.22E-25	Small nuclear ribonucleoprotein D3
nad3	−4.18	8.12	1.15E-22	Nad3
AFUN005575	−2.96	4.91	1.03E-21	Estrogen receptor binding site associated antigen 9 variant 1
AFUN008128	−2.06	5.96	9.59E-19	Transcription initiation factor TFIIF subunit alpha
AFUN005032	−1.86	6.56	6.57E-18	mRNA turnover protein 4
AFUN014198	−2.51	9.41	1.94E-16	40S ribosomal protein S29
AFUN011059	−2.05	5.27	6.44E-15	-
AFUN001216	−1.94	5.18	1.12E-14	-
AFUN006762	−2.43	9.41	4.03E-14	-
AFUN009976	−1.82	6.48	9.17E-14	Nuclear protein NHN1
AFUN014520	−2.04	5.27	9.09E-13	CCR4-NOT transcription complex subunit 7/8
AFUN014691	−1.91	5.50	9.63E-13	-
AFUN002476	−3.32	6.87	2.21E-12	-
nad6	−2.65	8.06	2.44E-12	Nad6
AFUN009128	−1.69	5.29	2.86E-12	Nat13 protein
AFUN007527	−2.88	2.97	5.92E-12	Phosphopantothenoylcysteine decarboxylase
AFUN005236	−1.84	8.05	6.25E-12	40S ribosomal protein S21
AFUN003657	−1.77	5.92	6.78E-12	-
AFUN010447	−2.04	4.37	1.08E-11	26 proteasome complex subunit DSS1

^a^Log_2_ fold change between ‘expression’ levels in Ribo-Zero and poly(A) samples (for values >0, Ribo-Zero > poly(A); for values <0, poly(A) > Ribo-Zero)

^b^Log_2_ counts per million mapped reads. Mean ‘expression’ level for all samples

^c^Significance of differential ‘expression’, *P*-value adjusted for a 5 % false discovery rate

**Fig. 3:**
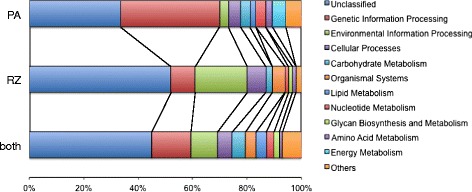
Representation of functional categories in poly(A) and Ribo-Zero over-represented gene sets and in genes not differentially represented (“both”)

**Fig. 4:**
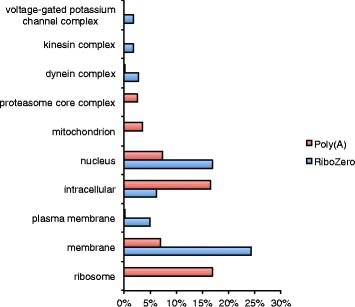
Cellular compartment GO terms differentially represented between gene sets over-represented in poly(A) (red) and Ribo-Zero (blue) samples

A broader analysis of functional categories and gene ontologies represented in the gene sets showed differences between poly(A) and Ribo-Zero libraries. The broad functional categories (as defined in the KEGG databases) of genes over-represented in the Ribo-Zero or poly(A) libraries were quite different. 28 % (516/1,841) of genes were annotated for the Ribo-Zero set, compared to 51 % (643/1,250) for the poly(A) set and 36 % (3702/10,253) for genes not differentially represented among treatments. The largest category (after “unclassified”) for the Ribo-Zero set was “environmental information processing”, while for the poly(A) set it was “genetic information processing” (Fig. 3). Many of these “genetic information processing”-associated genes in the poly(A) set were ribosomal proteins, as indicated by gene ontology analysis (summarised in Table 5), where ribosome-associated terms were enriched in this set (Fig. 4 and Additional file 1). In contrast, many of the “environmental information processing”-associated genes in the Ribo-Zero set may be membrane and/or nuclear proteins involved in signal transduction or transcriptional regulation (Fig. 4 and Additional file 1).

**Table 5:** Summary of gene ontology analysis

GO domain	Instances^a^ (BG^b^)	Instances (RZ^b^)	Instances (PA^b^)	RZ *vs.* BG^c^	PA *vs.* BG^c^	RZ *vs.* PA^c^
Biological process	7,228	763	711	11	9	19
Molecular function	14,362	1,912	1,109	15	13	25
Cellular compartment	3,648	403	515	4	10	10

^a^An “instance” refers to a GO term associated with a gene ID

^b^BG = background (whole genome); RZ = Ribo-Zero; PA = poly(A)

^c^Significantly differentially represented GO terms (adjusted *p* < 0.05)

### Detection of un-annotated transcripts

**Fig. 5:**
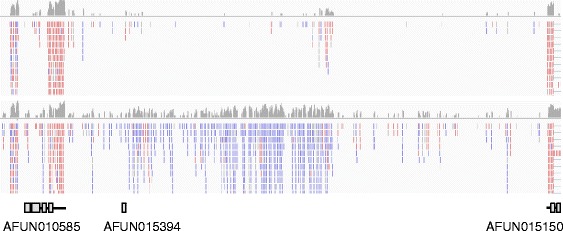
Example of putative novel transcript(s) represented in the Ribo-Zero libraries but not in the poly(A) libraries. The top panel shows a coverage depth summary plot (grey plot; log-scaled coverage depth) and aligned reads (red bars are reads from pairs aligned to the plus strand, blue bars from pairs aligned to the minus strand) for the poly(A) data. Below these are the same for the Ribo-Zero data. Below these are gene models (grey boxes represent exons, black lines un-translated regions: introns and UTRs) from the gene set AfunF1.2. A large transcribed region on the minus strand is evident in the Ribo-Zero data only, representing one or more putatively poly(A)- transcripts

To further compare the transcriptomes, transcripts were annotated from aligned data from each method. 11,395 genes (15,605 transcripts) were predicted using the poly(A) data and 15,914 genes (20,317 transcripts) using the Ribo-Zero data (Table 6). After removing transcripts that overlapped those predicted in the AfunF1.2 annotation, more remained in the Ribo-Zero transcriptome (nearly 5,000) that in the poly(A) transcriptome (around 1,000), suggesting the presence of transcripts not detected in the poly(A) transcriptome. Some of this difference could be due to an increased amount of immature transcripts (that are neither fully spliced nor polyadenylated) and/or a greater contribution from DNA contamination in the Ribo-Zero samples (which are subjected to less PCR amplification specifically of transcripts). However, visual inspection of a sample of these putative transcripts showed that a number were indeed unannotated, Ribo-Zero-specific transcribed regions that did not occur within the introns of annotated genes and were predominantly strand-specific (i.e. did not result from DNA contamination, which would not be strand-specific). An example is shown in Fig. 5. Further analyses are needed to elucidate the functions of these transcripts.

**Table 6:** Comparison of the published genome annotation with *de novo* annotations based on RNAseq alignments

	AfunF1.2^a^	Poly(A) (not in ref^b^)	Ribo-Zero (not in ref^b^)
Scaffolds with annotation	622	587 (415)	727 (645)
Genes	13,757	11,395 (1,000)	15,914 (4,744)
Transcripts	13,898	15,605 (1,039)	20,317 (4,831)

^a^Excludes mitochondrial genome

^b^Features that do not overlap any in the reference annotation

### Validation and improvement of the annotation of *Anopheles funestus* genes, focused on detoxification-associated gene families

**Fig. 6:**
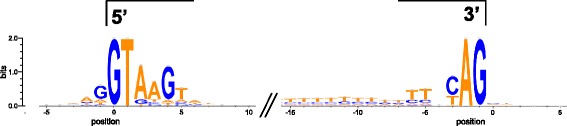
Consensus sequences at the 5′ and 3′ intron-exon boundaries of 28,346 introns validated by RNAseq. Position 0 at the 5′ end marks the first nucleotide in the intron. Position 0 at the 3′ end marks the first nucleotide in the following exon. Two diagonal lines denote the variable central portions of the introns. The y-axis indicates the bit-score for each nucleotide position. An extended motif of GTAAGT is seen at the 5′ end, with a slight enrichment of purines at the last position of the preceding exon. The conserved AG motif is seen at the 3′ end, preceded by an extended pyrimidine-rich region. Little or no sequence conservation is seen in the flanking exons, apart from at the last position of the preceding exon

An important part of genome annotation is validation of the predicted gene models. These RNAseq data allowed this to be done. On a genome-wide scale there were 44,031 predicted introns, of which 43,852 were unique (i.e. not the same intron in multiple transcript isoforms or overlapping genes). Of these, 28,346 (61.64 %) were validated by at least 10 reads in the FANG-1, 2 and 3 poly(A) data sets (31,927 introns, 72.91 %, were validated by at least 1 read in the same data set). A small number of introns were supported at only one end, suggesting mis-annotation of the gene models. Of the 44,031 predicted introns, 1,125 were supported only at the 5′ end and 2,118 only at the 3′ end. Introns supported only at the 3′ end tend to be the first predicted intron (45 % the first intron *vs.* 13 % the last intron), while introns supported only at the 5′ end tend to be the last predicted intron (33 % the last intron *vs.* 17 % the first intron), suggesting that the ends of up to around 3,000 gene models are mis-annotated and could be manually improved. Results of the validation analysis for all predicted introns are shown in Additional file 3. For the 28,346 validated introns, consensus motifs at intron-exon boundaries are shown in Fig. 6. The 5′ end of the intron has an extended GTAAGT motif, while the 3′ end of the intron has the conserved AG motif preceded by an extended pyrimidine-rich region. No conserved motif is seen in the flanking exons, except for a slight enrichment of purines at the last position of the upstream exon.

**Fig. 7:**
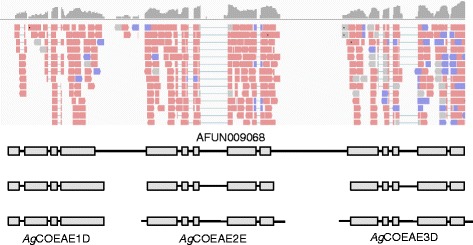
Example of an incorrect gene model (AFUN009068) and three manually edited gene models. The top panel shows a coverage depth summary plot (grey plot; log-scaled coverage depth) and aligned reads (red bars are reads from pairs aligned to the plus strand, blue bars from pairs aligned to the minus strand). Below these are the gene models (grey boxes represent exons, black lines un-translated regions: introns and UTRs). The gene model in gene set AfunF1.2 (AFUN009068) is shown above three manually edited gene models based on read alignments and orthologous genes in the *Anopheles gambiae* (*Ag*) genome annotation (bottom). The three genes are carboxylesterases (COE)

Visual inspection of the automated MAKER genome annotation of *Anopheles funestus* showed that, in many cases, the MAKER annotation had inferred a single gene model from multiple closely located genes. This behaviour is particularly problematic for detoxification-associated genes such as cytochrome P450 and GST because these genes often occur in clusters of tandemly duplicated paralogous genes. Manual editing was required to improve the genome annotation, an example of which is shown in Fig. 7.

We aimed to improve the annotation of genes involved in the three phases of detoxification: transformation (phase I), conjugation (phase II) and transport/efflux (phase III). Two major enzyme families, P450 (phase I) and GST (phase I and II), have already been manually curated and, at the time of writing, are being incorporated into the VectorBase genome annotation, AfunF1.3 (Dr. Craig Wilding and Dr. Dan Lawson, personal communications). Therefore, we focused upon seven other detoxification gene families. These were: the phase I enzymes carboxylesterases (COE), flavin-containing monooxygenases (FMO), aldehyde oxidases (AOX) and aldehyde dehydrogenases (ALDH); the phase II enzymes UDP glucosyltransferases (UGT) and sulfotransferases (SULT); and the phase III transporter-encoding ATP-binding cassette genes (ABC).

Manual editing considerably improved the annotation of detoxification-associated genes. A table of manually edited gene models and unedited, putatively correct, gene models belonging to each family is shown in Additional file 4.

For the carboxylestarase genes (COE), 22 gene models were manually edited to produce 28 putative COE genes and 6 non-COE genes, 10 gene models were not modified. For the flavin-containing monooxygenase genes (FMO) 4 gene models were identified and were not modified. For the aldehyde oxidase genes (AO), 9 gene models were manually edited to produce 6 putative AO genes, 2 xanthine dehydrogenase genes and 2 non-AO genes, 2 gene models were not modified. For the aldehyde dehydrogenase genes (ALDH), 2 gene models were manually edited to produce 2 ALDH genes and 1 non-ALDH gene, 1 gene model was not modified.

For the UDP-glycosyltransferase genes (UGT), 4 gene models were manually edited to produce 8 UGT genes and 3 non-UGT genes, 14 gene models were not modified. For the sulfotransferase genes (SULT), 1 gene model was manually edited to produce 5 SULT genes and and an oestrogen sulfotransferase gene, 3 gene models were not modified.

For the ATP-binding cassette genes (ABC), 9 gene models were manually edited to produce 13 ABC genes and 10 non-ABC genes, 16 gene models were not modified.

All manually edited gene models have been submitted to VectorBase for incorporation into the *Anopheles funestus* genome annotation.

### Integration of *An. funestus* transcriptome datasets and assignment of FUMOZ scaffolds to chromosome arms

Two *An. funestus* whole transcriptome datasets were published before the whole genome assembly [22, 23]. We integrated both the “Crawford” transcriptome (14,850 sequences) and the “Gregory” transcriptome (18,103 sequences) with the FUMOZ predicted transcript set, AfunF1.2 (13,897 sequences) by clustering them based on sequence similarity. The results are shown in Additional file 5. Clustering the full set of 46,850 sequences at 95 % similarity resulted in 25,519 clusters. The results suggested that the “Gregory” transcriptome was more fragmented than the other two: its 18,103 sequences were represented in only 5,741 unique clusters, compared to 12,349 clusters for the “Crawford” transcriptome and 13,172 clusters for the AfunF1.2 transcript set.

Genome sequences assembled to the level of whole chromosomes are more useful than draft assemblies for analyses of genome wide variation and gene expression, because structural and spatial information can be important (e.g. for defining the location of a selective sweep or the effect of regional chromatin remodelling on co-regulation of gene expression). The comparison of *An. funestus* predicted genes to those of *An. gambiae* allowed a number of scaffolds to be tentatively assigned to chromosome arms based on homology to *An. gambiae*. Table 7 summarises the number of scaffolds with 5 or more genes assigned to a chromosome arm by putative 1:1 orthology with *An. gambiae* (all scaffold IDs are listed in Additional file 6). In a small number of anomalous cases where a scaffold contained genes with orthology to genes on a different chromosome *An. gambiae* arm, the minority chromosome arm was never supported by more than 1 gene and these were removed by setting a threshold of 5 genes to support assignment of a scaffold to a group. This conservative approach to defining linkage groups assigned only a small number (286/1392 = 20.55 %) of scaffolds. However, these were generally large scaffolds and represented the greater proportion (183,517,537/225,223,604 = 81.48 %) of the total genome length. These groups could form a starting point for genome assembly finishing and for further analysis of synteny and co-linearity of genes between *An. funestus* and *An. gambiae*.

**Table 7:** *Anopheles funestus* scaffolds assigned to groups representing putative chromosome arms

Chromosome arm ^a^	Scaffolds ^b^	Total length (bp) ^c^
X	27	16,915,574
2R	78	52,607,791
2 L	57	42,990,663
3R	72	41,849,609
3 L	52	29,153,900
Total	286	183,517,537

^a^ *An. funestus* chromosome arm, accounting for the whole arm translocation between 2 L and 3R relative to *An. gambiae*

^b^ Number of *An. funestus* scaffolds with 5 or more putative 1:1 orthologues with *An. gambiae*

^c^ Length includes sequencing gaps

## Conclusions

The sequencing of the genome of the major malaria vector *Anopheles funestus* allows new analyses of its biology and may accelerate research on this species, which currently lags behind that of *Anopheles gambiae*. To carry out analyses of gene expression relevant to insecticide resistance, we have piloted the use of RNAseq. In addition, we have improved the genome annotation for a number of gene families with roles in the detoxification of xenobiotic agents.

As reported in other eukaryotic systems, the method used to enrich for mRNA relative to rRNA has a large effect upon the observed transcriptome [18–21]. Functional classes of genes are differentially represented in poly(A) mRNA-enriched and Ribo-Zero rRNA-depleted transcriptomes, with greater representation of ribosomal proteins with poly(A) and membrane-associated proteins with Ribo-Zero. Genes with both poly(A) + and poly(A)- transcripts, such as histones, and ncRNA, such as signal recognition particle RNAs, were also highly represented in the Ribo-Zero transcriptome. The Ribo-Zero libraries show a higher proportion of reads aligning outside of annotated exons, which may represent a combination of novel, poly(A)- transcripts and the introns of immature transcripts.

In making recommendations for which method to use for studies of the transcriptomes of mosquitoes, several factors should be considered. For analyses of differential gene expression, power to detect different expression levels is proportional to the number of reads aligned to each gene. Poly(A) mRNA enrichment does provide more of this ‘on-target’ data than Ribo-Zero rRNA depletion, yet the difference is less striking after accounting for rRNA genes, in particular a highly poly(A)-enriched mitochondrial 16S rRNA. The transcriptome profiles of poly(A) samples were more highly correlated with each other than Ribo-Zero samples, indicating that power to detect differential gene expression would be greater for poly(A) mRNA-enriched samples. However, we argue that Ribo-Zero rRNA depletion produces a truer representation of the transcriptome because (i) there is less PCR amplification of transcripts, which can bias the final results and (ii) The transcriptome includes the poly(A)- fraction, which may be functionally relevant. Due to the large effect that the mRNA enrichment method has on the transcriptome obtained, researchers should be very careful in comparing results obtained using different methods.

The draft genome annotation of *An. funestus* is currently inadequate for detailed studies of gene expression targeted at insecticide resistance mechanisms. Major gene families involved in detoxification of xenobiotic substances, such as cytochrome P450 and GST genes, are not well annotated by automated approaches. Manually edited gene models for these gene families are being incorporated into the genome annotation at the time of writing (Dr. Craig Wilding and Dr. Dan Lawson, personal communications). We extended this annotation improvement to 7 more key detoxification genes families: 4 associated with phase I; 2 with phase II and 1 with phase III. This effort offers a major improvement of the utility of the *An. funestus* genome as a tool to study insecticide resistance in this species. Accurate annotation of these gene families is particularly important for *An. funestus*, as insecticide resistance appears to be primarily mediated by detoxification of the compounds in this species.

## Methods

### Mosquito samples used in the study

The study used *Anopheles funestus* mosquitoes of the FANG laboratory colony, a fully insecticide susceptible colony derived from Angola [28]. Mosquito eggs (a kind gift of Prof. Lizette Koekemoer, University of the Witwatersrand, South Africa) were hatched and mosquitoes reared to adulthood in the insectaries at the Liverpool School of Tropical Medicine under conditions described elsewhere [2].

### RNA extraction, sequence library preparation and sequencing

Total RNA was extracted from three pools of 10 individual adult female mosquitoes using the Arcturus PicoPure RNA isolation kit (Life Technologies, Carlsbad, USA), according to the manufacturer’s instructions and including a DNase treatment step. On visual inspection of the purified RNA, the samples displayed some pigmentation, the likely effect of which on data quality was unknown. To assess this, the most abundant sample (FANG-1) was split in two. One half of the sample was cleaned using Agencourt AMPure XP beads (Beckman Coulter, Brea, USA) prior to further processing.

All four samples were split in two and one of each pair subjected to poly(A) mRNA enrichment and the other to ribosomal RNA depletion. Polyadenylated RNA was selected from total RNA samples using 3 rounds of poly(A) selection with the Dynabeads mRNA purification kit (Life Technologies), using 1.5 μg of starting material. Total RNA was rRNA-depleted using the Ribo-Zero low input kit for Human/Mouse/Rat (Illumina, San Diego, USA), using 100 ng of starting material. RNAseq libraries were prepared from poly(A) and Ribo-Zero mRNA-enriched material using the ScriptSeq v2 RNAseq library preparation kit (Illumina), using 15 cycles of PCR amplification. Libraries were purified using Agencourt AMPure XP beads (Beckman Coulter). Each library was quantified using a Qubit fluorometer (Life Technologies) and the size distribution assessed using the 2100 Bioanalyzer (Agilent Technologies, Santa Clara, USA).

The eight final libraries were pooled in equimolar amounts using the Qubit and Bioanalyzer data. The quantity and quality of each pool was assessed by Bioanalyzer and subsequently by qPCR using the Kapa Illumina library quantification kit (Kapa Biosystems, Wilmington, USA), on a Light Cycler LC480II (Roche, Basel, Switzerland), according to manufacturers’ instructions. The pool of libraries was sequenced on one lane of the HiSeq 2500 (Illumina) at 2x125 bp paired-end sequencing with v4 chemistry. Sequence library preparation and sequencing were done at the Centre for Genomic Research, University of Liverpool, UK.

### Alignment of sequence reads to a reference genome

Initial processing and quality assessment of the sequence data was performed using a software pipeline developed at the Centre for Genomic Research, University of Liverpool (Dr. Richard Gregory, personal communication). In this pipeline, basecalling and de-multiplexing of indexed reads was performed by CASAVA version 1.8.2 (Illumina) to produce samples from the pooled sequence data, in fastq format. The raw fastq files were trimmed to remove Illumina adapter sequences using Cutadapt version 1.2.1 [29]. The 3' end of any read matching the adapter sequence over at least 3 bp was trimmed off. Reads were further trimmed to remove low quality bases, using Sickle version 1.200 [30], with a minimum window quality score of 20. After trimming, reads shorter than 10 bp were removed. If both reads from a pair passed this filter, each was included in either the R1 (forward reads) or R2 (reverse reads) file. If only one of a read pair passed this filter, it was included in the R0 (unpaired reads) file.

The reference sequence used for alignment was the *Anopheles funestus* assembled scaffold sequences derived from the FUMOZ laboratory colony, a multi-insecticide resistant colony derived from southern Mozambique [11, 28]. Assembly AfunF1 (GenBank assembly identifier GCA_000349085.1; GenBank WGS project identifier APCI01) was downloaded from VectorBase [12, 13]. A single sequence representing the *Anopheles funestus* mitochondrial genome (accession number DQ146364) [31] was added to these scaffolds to make the reference sequence used in the analysis.

R1/R2 read pairs were aligned to the reference sequence using TopHat version 2.0.10 [32], based on the bowtie2 aligner [33]. The alignment was carried out using the following non-default parameters: the insert inner distance (between 3′ ends of R1 and R2 reads) was set to 0, with a standard deviation of 50 (options “--mate-inner-dist 0” and “--mate-std-dev 50”); for reads with multiple alignments in the genome(s), only a single, best alignment was recorded, with ties assigned randomly (option “--max-multihits 1”); the library type was specified as second-stranded (option “--library-type fr-secondstrand”), as it was a ScriptSeq library (in second strand sequencing, the forward read of a pair, R1, will map sense to a transcript, and the reverse read, R2, will map antisense).

### Analysis of transcript abundance among samples

Tags mapped in the sense orientation to annotated *Anopheles funestus* genes (automated predictions from gene set AfunF1.2, 2014-08-22, downloaded from VectorBase and annotated genes from the mitochondrial genome) were counted using htseq-count, part of the ‘HTSeq’ framework, version 0.5.3p9 [34]. Read count data were analysed in the R environment, in particular using the package edgeR for differential gene expression analysis [35].

Analysis of the tag count data used log_2_ transformed counts (after replacing 0 values with 1 s). Pairwise scatter plots of these values were plotted and the Pearson’s correlation coefficients (*r*^*2*^) represented in a sample correlation heatmap. Principal component analysis (PCA) was applied and the first, second and third principal components of variation plotted.

Differential gene expression analysis was carried out using edgeR [35]. Due to the low level of technical variation between cleaned and uncleaned FANG-1 samples, differential gene expression analysis used the mean tag count values for cleaned and uncleaned FANG-1 samples for each enrichment method. Two groups of samples (“Ribo-Zero” and “poly(A)”) were defined. Normalisation factors were calculated to correct for differences in total tag counts among samples, which may otherwise cause bias in differential gene expression analysis, using the “TMM” (Trimmed Mean M-values) method in edgeR [36] with default parameters. Common, trended (with a minimum of 500 genes in each bin) and tag-wise dispersion parameters were estimated. Trended dispersion was used for significance testing. Variation of RNAseq data can be modelled by a negative binomial distribution [37] and the data modelled using a generalized linear model [38]. For the contrast “Ribo-Zero / poly(A)”, the estimated log_2_ Fold Change for each gene was tested in edgeR using a Likelihood-Ratios (LR) test [39]. *P*-values associated with logFC were adjusted for multiple testing using the False Discovery Rate (FDR) approach [40]. Significantly differentially expressed genes were defined as those with an FDR-adjusted *P*-value < 5 %.

### Analysis of gene ontologies and KEGG functional categories

All GO terms associated with features in genome annotation AfunF1.2 were obtained from VectorBase. The terms were separated by GO domain: biological process (BP; 7,228 instances), molecular function (MF; 14,362 instances) and cellular compartment (CC; 3,648 instances). An “instance” refers to a GO term associated with a gene ID and the term is used here because multiple GO terms can be associated with the same gene ID and multiple gene IDs with the same GO term. To test for significant differences in GO term representation among sets of genes, Fisher’s exact test was applied for each GO term, with a correction for multiple testing using the method of Benjamini and Hochberg [40]. The gene sets tested were those over-represented in Ribo-Zero or in poly(A) libraries (with adjusted *p*-values < 0.01 in the test for differential expression).

Proteins were annotated using blastKOALA v2.0 [41], a tool available via the Kyoto Encyclopedia of Genes and Genomes, KEGG [42–44]. Four sets of proteins were submitted to blastKOALA: those over-represented in Ribo-Zero libraries (*n* = 1841); those over-represented in poly(A) libraries (*n* = 1250); those not significantly differentially represented (two sets, as a maximum of 10,000 sequences can be submitted at a time: *n* = 5531 and *n* = 4722). The protein sequence files were uploaded and the taxonomy ID of *An. funestus* (taxid:62324) entered. The database searched against was the “genus_eukaryotes” database (containing 3,696,044 entries).

### Detection of un-annotated transcripts

Alignment files of the four poly(A) mRNA-enriched libraries and the four Ribo-Zero rRNA-depleted libraries (in bam format) were merged to create a single poly(A) and a single Ribo-Zero alignment. Alignments to the mitochondrial genome were filtered out. These filtered alignments were used to predict transcripts using Cufflinks v2.2.1 [45]. Default parameters were applied except for the options “--library-type” (set to “fr-secondstrand”) and “--min-frags-per-transfrag” (set to 100 rather than the default 10). Putative transcripts were compared to the genome annotation using Cuffmerge and transcripts not associated with annotated features extracted for each of the poly(A) and the Ribo-Zero alignments. These transcripts were merged using Cuffmerge and the resulting file of features used in conjunction with the alignment files to generate read counts for poly(A) and the Ribo-Zero samples using featureCounts v1.4.6 [46].

### Genome annotation validation and improvement

Genomic location information for introns annotated in *An. funestus* gene set AfunF1.2 and the mitochondrial genome was extracted from the genome annotation. Similar location information for introns identified by alignment of RNAseq data, for the poly(A) samples, was extracted and the two datasets were compared to identify introns validated by RNAseq. Similar analyses were done for 5′ and 3′ intron ends alone. For validated introns, sequences spanning the 5′ and 3′ intron ends were extracted and used to identify enriched nucleotide motifs, represented as sequence logos using WebLogo 3 [47, 48].

Manual genome annotation improvement was carried out using *Anopheles gambiae* gene models as a reference. Starting with translated *An. gambiae* genes, BLASTp similarity searches were carried out in VectorBase to define the closest matching *An. funestus* proteins and identify gross differences in protein length. Then, gene models were compared and *An. funestus* gene models modified to match those of *An. gambiae* where appropriate. RNAseq alignments were used to guide the *An. funestus* gene model editing. As transcript ends are difficult to define from RNAseq alignments (due to coverage drop off near to molecule ends), edited gene models included putative protein coding regions but not putative un-translated regions. Edited gene models were submitted to VectorBase [12, 13].

### Clustering of sequences from multiple transcriptome datasets and assignment of scaffolds to chromosome arms

Sequences from two transcriptome assemblies were downloaded from NCBI [49]: the “Crawford” transcriptome, comprising 14,850 sequences (accessions EZ966136-EZ980985) and the “Gregory” transcriptome, comprising 18,103 sequences (accessions EZ915182-EZ933284). Sequences from the FUMOZ predicted transcript set AfunF1.2, comprising 13,897 sequences, were downloaded from VectorBase [12, 13]. The three datasets were concatenated and clustered by sequence identity using CD-HIT-EST v4.6 [50], with a clustering threshold of 95 % nucleotide identity.

To tentatively assign *An. funestus* genome assembly scaffolds to chromosome arms, predicted protein sequences from An. funestus (gene set Afun1.2) and *An. gambiae* (gene set Agam4.3) were downloaded from VectorBase [12, 13]. Sequence headers were parsed to retain the scaffold/chromosome information and the two datasets concatenated and clustered by peptide sequence identity using CD-HIT v4.6 [50], with a clustering threshold of 75 % amino acid identity. *An. funestus* scaffolds were assigned to a chromosome arm if they contained at least 5 putative 1:1 *An. gambiae* orthologues from that chromosome arm. Chromosome arms 2 L and 3R were switched to account for a whole arm translocation between 2 L and 3R in *An. funestus* relative to *An. gambiae*.

## Availability of supporting data

The RNAseq read data reported in this study were submitted to the European Nucleotide Archive (ENA) under the study accession PRJEB10294 (http://www.ebi.ac.uk/ena/data/view/PRJEB10294) and the sample accessions ERS809802-ERS809809.

## Additional files

Additional file: 1:
**Detailed and extra information and figures.** File containing detailed descriptions of the analyses and figures not included in the main article. (PDF 3540 kb) Additional file 2:
**Differential gene representation analysis results.** Table of results of differential gene representation analysis (for gene set Afun1.2 and mitochondrial genes) contrasting Ribo-Zero and poly(A) enriched RNAseq libraries. The most over-represented genes in Ribo-Zero libraries are highlighted in red. The most over-represented genes in poly(A) libraries are highlighted in blue. The significance cutoff used was an FDR-adjusted (FDR = 5 %) *P*-value < 0.01. The tag counts for FANG-1 samples are the mean of two libraries (see main text). (XLSX 1784 kb) Additional file 3:
**Validation of annotated exon-exon junctions.** Table of exon-exon junctions annotated in gene set Afun1.2 and the mitochondrial genome that were, or were not, validated by RNAseq data generated in this study. Introns validated at both ends (labelled 5′_and_3′) or at only one end (labelled 5′_only or 3′_only) are indicated. (XLSX 1949 kb) Additional file 4:
**Annotation improvements.** Table of gene annotation validation and/or manual improvement of *Anopheles funestus* detoxification gene models. Gene models for genes involved in the detoxification of xenobiotics were checked against both *Anopheles gambiae* orthologues and RNAseq alignments and modified where necessary. (XLSX 40 kb) Additional file 5:
**Integration of transcriptome datasets.** Table of sequence clusters (clustered at 95 % sequence identity) comprising AfunF1.2 gene set transcripts (13,897 sequences), the “Crawford” assembled transcriptome (14,850 sequences) and the “Gregory” assembled transcriptome (18,103 sequences). Each row represents a cluster. Sequences in a cluster that come from the same dataset are separated by commas. (XLSX 868 kb) Additional file 6:
**Scaffold assignment to chromosome arms.** Table of *Anopheles funestus* scaffolds assigned to chromosome arms. Assignment is based on the possession of at least 5 putative 1:1 orthologues with *Anopheles gambiae* occurring on the same chromosome arm. Orthology was inferred by clustering predicted protein sequences at 75 % amino acid identity. (XLSX 43 kb)

## Abbreviations

NGS: Next-generation sequencing
PCA: Principal component analysis
DGE: Differential gene expression
TMM: Trimmed mean M-values
LR: Likelihood ratio
FDR: False discovery rate
UTR: Un-translated region
GO: Gene ontology
RZ: Ribo-Zero
PA: Poly(A)
P450: Cytochrome P450 monooxygenase
GST: Glutathione S-transferase
COE: Carboxylesterase
FMO: Flavin-containing monooxygenase
AOX: Aldehyde oxidase
ALDH: Aldehyde dehydrogenase
UGT: UDP glucosyltransferase
SULT: Sulfotransferase
ABC: ATP-binding cassette transporter

## References

[1] Sinka ME, Bangs MJ, Manguin S, Coetzee M, Mbogo CM, Hemingway J (2010). The dominant Anopheles vectors of human malaria in Africa, Europe and the Middle East: occurrence data, distribution maps and bionomic précis. Parasites Vectors.

[2] Morgan JC, Irving H, Okedi LM, Steven A, Wondji CS (2010). Pyrethroid resistance in an *Anopheles funestus* population from Uganda. PLoS One.

[3] Wondji CS, Coleman M, Kleinschmidt I, Mzilahowa T, Irving H, Ndula M (2012). Impact of pyrethroid resistance on operational malaria control in Malawi. Proc Natl Acad Sci U S A.

[4] Riveron JM, Irving H, Ndula M, Barnes KG, Ibrahim SS, Paine MJ (2013). Directionally selected cytochrome P450 alleles are driving the spread of pyrethroid resistance in the major malaria vector *Anopheles funestus*. Proc Natl Acad Sci U S A.

[5] Riveron JM, Yunta C, Ibrahim SS, Djouaka R, Irving H, Menze BD (2014). A single mutation in the GSTe2 gene allows tracking of metabolically based insecticide resistance in a major malaria vector. Genome Biol.

[6] Riveron JM, Ibrahim SS, Chanda E, Mzilahowa T, Cuamba N, Irving H (2014). The highly polymorphic CYP6M7 cytochrome P450 gene partners with the directionally selected CYP6P9a and CYP6P9b genes to expand the pyrethroid resistance front in the malaria vector *Anopheles funestus* in Africa. BMC Genomics.

[7] Choi KS, Christian R, Nardini L, Wood OR, Agubuzo E, Muleba M (2014). Insecticide resistance and role in malaria transmission of *Anopheles funestus* populations from Zambia and Zimbabwe. Parasites Vectors.

[8] Mulamba C, Riveron JM, Ibrahim SS, Irving H, Barnes KG, Mukwaya LG (2014). Widespread pyrethroid and DDT resistance in the major malaria vector *Anopheles funestus* in East Africa is driven by metabolic resistance mechanisms. PLoS One.

[9] Glunt KD, Abílio AP, Bassat Q, Bulo H, Gilbert AE, Huijben S (2015). Long-lasting insecticidal nets no longer effectively kill the highly resistant *Anopheles funestus* of southern Mozambique. Malar J.

[10] Holt RA, Subramanian GM, Halpern A, Sutton GG, Charlab R, Nusskern DR (2002). The genome sequence of the malaria mosquito *Anopheles gambiae*. Science.

[11] Neafsey DE, Waterhouse RM, Abai MR, Aganezov SS, Alekseyev MA, Allen JE (2015). Mosquito genomics. Highly evolvable malaria vectors: the genomes of 16 Anopheles mosquitoes. Science.

[12] Giraldo-Calderón GI, Emrich SJ, MacCallum RM, Maslen G, Dialynas E, Topalis P (2015). VectorBase: an updated bioinformatics resource for invertebrate vectors and other organisms related with human diseases. Nucleic Acids Res.

[13] VectorBase. https://www.vectorbase.org

[14] Cantarel BL, Korf I, Robb SM, Parra G, Ross E, Moore B (2008). MAKER: an easy-to-use annotation pipeline designed for emerging model organism genomes. Genome Res.

[15] Holt C, Yandell M (2011). MAKER2: an annotation pipeline and genome-database management tool for second-generation genome projects. BMC Bioinformatics.

[16] Djebali S, Davis CA, Merkel A, Dobin A, Lassmann T, Mortazavi A (2012). Landscape of transcription in human cells. Nature.

[17] de Klerk E, ‘t Hoen PA (2015). Alternative mRNA transcription, processing, and translation: insights from RNA sequencing. Trends Genet.

[18] Cui P, Lin Q, Ding F, Xin C, Gong W, Zhang L (2010). A comparison between ribo-minus RNA-sequencing and polyA-selected RNA-sequencing. Genomics.

[19] Yang L, Duff MO, Graveley BR, Carmichael GG, Chen LL (2011). Genomewide characterization of non-polyadenylated RNAs. Genome Biol.

[20] Zhao W, He X, Hoadley KA, Parker JS, Hayes DN, Perou CM (2014). Comparison of RNA-Seq by poly (A) capture, ribosomal RNA depletion, and DNA microarray for expression profiling. BMC Genomics.

[21] Sultan M, Amstislavskiy V, Risch T, Schuette M, Dökel S, Ralser M (2014). Influence of RNA extraction methods and library selection schemes on RNA-seq data. BMC Genomics.

[22] Crawford JE, Guelbeogo WM, Sanou A, Traoré A, Vernick KD, Sagnon N (2010). *De novo* transcriptome sequencing in *Anopheles funestus* using Illumina RNA-seq technology. PLoS One.

[23] Gregory R, Darby AC, Irving H, Coulibaly MB, Hughes M, Koekemoer LL (2011). A *de novo* expression profiling of *Anopheles funestus*, malaria vector in Africa, using 454 pyrosequencing. PLoS One.

[24] Winnebeck EC, Millar CD, Warman GR (2010). Why does insect RNA look degraded?. J Insect Sci.

[25] Marzluff WF, Wagner EJ, Duronio RJ (2008). Metabolism and regulation of canonical histone mRNAs: life without a poly(A) tail. Nat Rev Genet.

[26] Kühl M, Wedlich D (1995). XB/U-cadherin mRNA contains cytoplasmic polyadenylation elements and is polyadenylated during oocyte maturation in *Xenopus laevis*. Biochim Biophys Acta.

[27] Beilharz TH, Preiss T (2007). Widespread use of poly(A) tail length control to accentuate expression of the yeast transcriptome. RNA.

[28] Hunt RH, Brooke BD, Pillay C, Koekemoer LL, Coetzee M (2005). Laboratory selection for and characteristics of pyrethroid resistance in the malaria vector *Anopheles funestus*. Med Vet Entomol.

[29] Martin M (2011). Cutadapt removes adapter sequences from high-throughput sequencing reads. EMBnet J.

[30] Joshi NA, Fass JN. Sickle. A sliding-window, adaptive, quality-based trimming tool for FastQ files (Version 1.33). https://github.com/najoshi/sickle. 2011

[31] Krzywinski J, Grushko OG, Besansky NJ (2006). Analysis of the complete mitochondrial DNA from *Anopheles funestus*: an improved dipteran mitochondrial genome annotation and a temporal dimension of mosquito evolution. Mol Phylogenet Evol.

[32] Kim D, Pertea G, Trapnell C, Pimentel H, Kelley R, Salzberg SL (2013). TopHat2: accurate alignment of transcriptomes in the presence of insertions, deletions and gene fusions. Genome Biol.

[33] Langmead B, Salzberg SL (2012). Fast gapped-read alignment with Bowtie 2. Nat Methods.

[34] HTSeq. http://www-huber.embl.de/users/anders/HTSeq/doc/count.html

[35] Robinson MD, McCarthy DJ, Smyth GK (2010). edgeR: a Bioconductor package for differential expression analysis of digital gene expression data. Bioinformatics.

[36] Robinson MD, Oshlack A (2010). A scaling normalization method for differential expression analysis of RNA-seq data. Genome Biol.

[37] Robinson MD, Smyth GK (2007). Moderated statistical tests for assessing differences in tag abundance. Bioinformatics.

[38] Nelder JA, Wedderburn RWN (1972). Generalized linear models. J R Stat Soc Ser A.

[39] Wilks SS (1938). The large-sample distribution of the likelihood ratio for testing composite hypotheses. Ann Math Stat.

[40] Benjamini Y, Hochberg Y (1995). Controlling the false discovery rate: a practical and powerful approach to multiple testing. J R Stat Soc Ser B.

[41] BlastKOALA. http://www.kegg.jp/blastkoala. Accessed April, 2015.

[42] Kanehisa M, Goto S (2000). KEGG: kyoto encyclopedia of genes and genomes. Nucleic Acids Res.

[43] Kanehisa M, Goto S, Sato Y, Kawashima M, Furumichi M, Tanabe M (2014). Data, information, knowledge and principle: back to metabolism in KEGG. Nucleic Acids Res.

[44] Kyoto Encyclopedia of Genes and Genomes (KEGG). http://www.genome.jp/kegg. Accessed April, 2015.

[45] Trapnell C, Williams BA, Pertea G, Mortazavi A, Kwan G, van Baren MJ (2010). Transcript assembly and quantification by RNA-Seq reveals unannotated transcripts and isoform switching during cell differentiation. Nat Biotechnol.

[46] Liao Y, Smyth GK, Shi W (2014). featureCounts: an efficient general purpose program for assigning sequence reads to genomic features. Bioinformatics.

[47] Schneider TD, Stephens RM (1990). Sequence logos: a new way to display consensus sequences. Nucleic Acids Res.

[48] Crooks GE, Hon G, Chandonia JM, Brenner SE (2004). WebLogo: a sequence logo generator. Genome Res.

[49] NCBI. http://www.ncbi.nlm.nih.gov

[50] Fu L, Niu B, Zhu Z, Wu S, Li W (2012). CD-HIT: accelerated for clustering the next-generation sequencing data. Bioinformatics.

