# Supplementary material for: Molecular tools for studying the major malaria vector Anopheles funestus: improving the utility of the genome using a comparative poly(A) and Ribo-Zero RNAseq analysis
Source: BMC Genomics. 2015 Nov 14;16:931. doi: 10.1186/s12864-015-2114-z (PMC4647341; doi:10.1186/s12864-015-2114-z)

Detailed and extra information and figures not included in the main text.

Preparation of transcriptome sequencing libraries

We aimed to compare two methods of mRNA-enrichment: ribosomal RNA depletion and poly(A)-selection of messenger RNA. Samples were assessed before and after mRNA enrichment. BioAnalyzer traces of total RNA and of RNA after ribosomal RNA depletion or poly(A)-selection of messenger RNA are shown for each biological replicate sample in Figures 1, 2 and 3. The total RNA traces (Figures 1A, 2A and 3A) show the two 28S subunits of similar size to the 18S rRNA rather than a major 28S rRNA peak, which invalidates the use of the RNA integrity number (RIN) to assess RNA quality. A smaller peak of less than 4000 bp in the total RNA samples may represent the intact 28S rRNA. In Figure 2 (FANG-2), there is an additional small peak of more than 4000 bp. It is possible that this represents genomic DNA contamination of the sample, which may contribute to the FANG-2 (Ribo-Zero) sample being divergent from FANG-1 and FANG-3 in later analyses based on its transcriptome profile. All of the samples subjected to poly(A)-selection showed a large peak at around 2000 bp. Based on later analyses of read counts, we suggest that this is a mitochondrial rRNA gene that is enriched by poly(A) selection.

All 8 Ribo-Zero and poly(A) samples were used to generate transcriptome sequencing libraries, prepared as described in the Methods section of the main text.

Figure 1. BioAnalyzer traces for sample FANG-1 of: (A) total RNA; (B) RNA after Ampure clean-up followed by Ribo-Zero rRNA depletion; (C) RNA after Ribo-Zero rRNA depletion; (D) RNA after Ampure clean-up followed by three rounds of poly(A) mRNA enrichment; (E) RNA after three rounds of poly(A) mRNA enrichment.

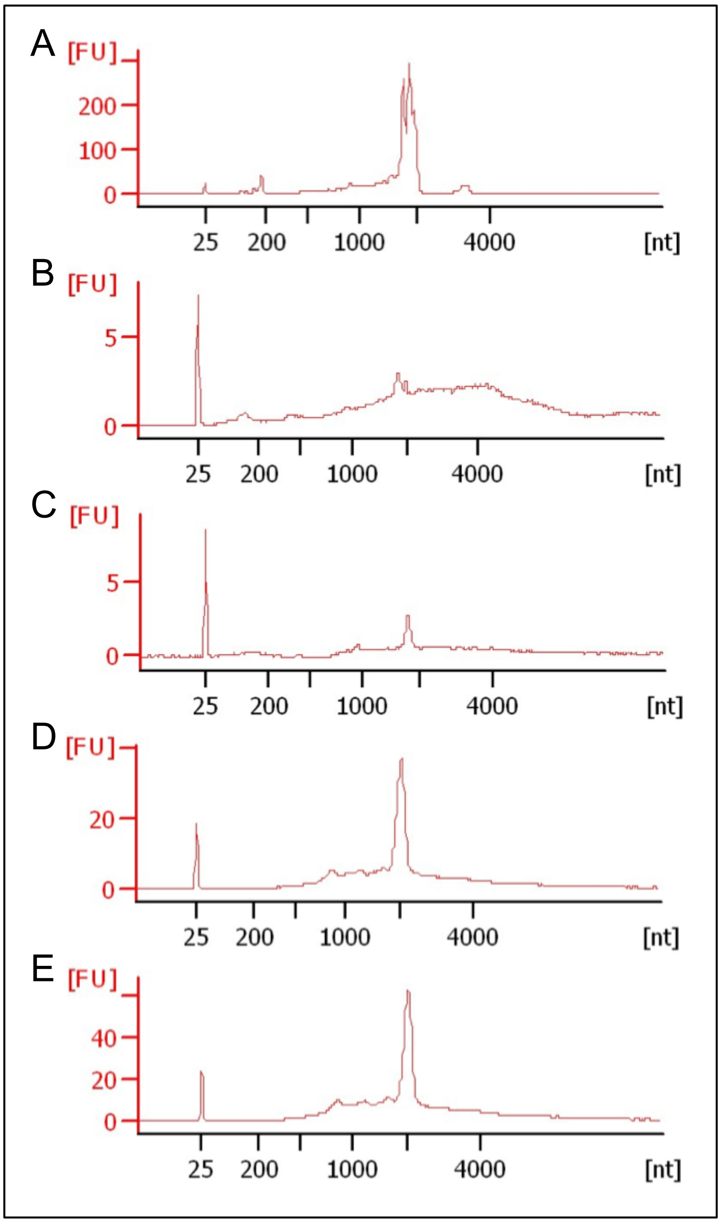

Figure 2. BioAnalyzer traces for sample FANG-2 of: (A) total RNA; (B) RNA after Ribo-Zero rRNA depletion; (C) RNA after three rounds of poly(A) mRNA enrichment.

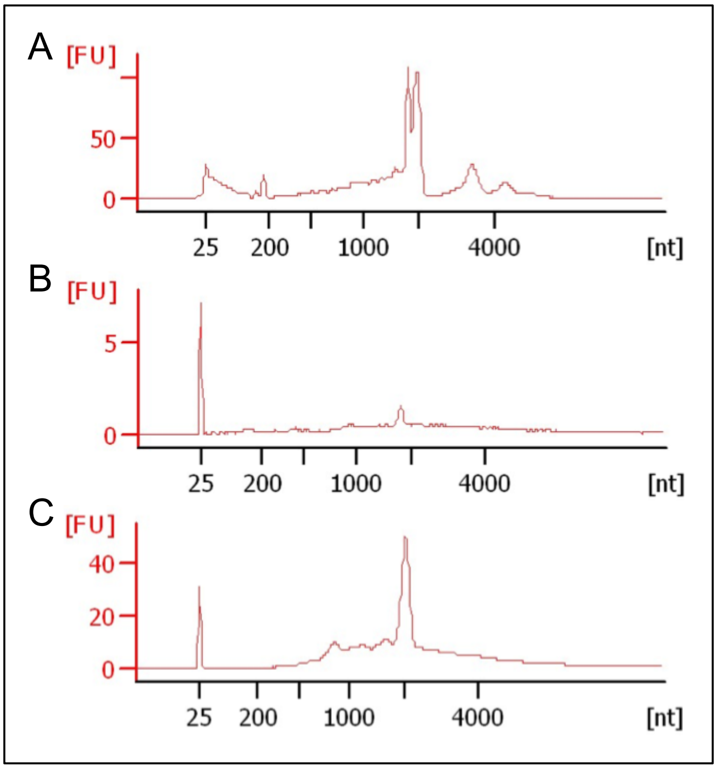

Figure 3. BioAnalyzer traces for sample FANG-3 of: (A) total RNA; (B) RNA after Ribo-Zero rRNA depletion; (C) RNA after three rounds of poly(A) mRNA enrichment.

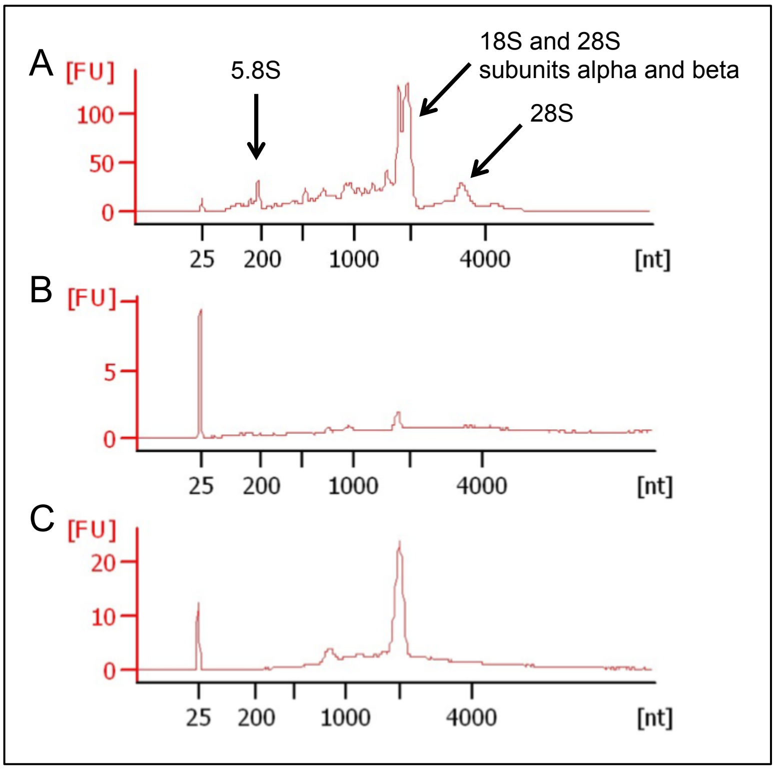

Analysis of sequence read data quality

After paired-end sequencing (2x125bp), the quality of the sequence data was assessed. Table 1 summarises the read counts before and after adapter and quality trimming. Sequencing produced a total of 481,922,366 reads (240,961,183 read pairs) for all libraries. The eight libraries were quite evenly represented in the pool, with a median of 61,229,870 reads per library (range 44,384,316 to 68,640,804 reads). Adapter and quality trimming and subsequent length filtering removed a small number of reads from each library (median 342,250 reads removed; range 270,230 to 392,794 reads removed). The numbers of unpaired reads after trimming were correspondingly low (less than 0.6% of the total trimmed reads in all cases). Figure 4 shows the read length distributions after adapter and quality trimming. Note that R0 (unpaired) reads are generally trimmed more than paired reads as they more often represent poor quality sequence. Based on these numbers, the quality of the sequence data was good.

Table 1. Descriptive statistics of sequenced libraries.

Sample ID	Sample ID (short)	Index	Untrimmed reads	Trimmed reads	R1/R2 pairs [1]	R0 reads (%) [2]
FANG1_cleanedRZ	F1_XP_RZ	CAGATC	68,640,804	68,248,010	33,947,721	352,568 (0.52%)
FANG1_cleaned_PolyA	F1_XP_PA	CTTGTA	66,165,202	65,876,491	32,802,659	271,173 (0.41%)
FANG1_uncleanedRZ	F1_RZ	TGACCA	61,161,962	60,814,246	30,239,816	334,614 (0.55%)
FANG1_uncleaned_polyA	F1_PA	GATCAG	64,207,516	63,870,732	31,777,725	315,282 (0.49%)
FANG2_uncleanedRZ	F2_RZ	ACAGTG	44,384,316	43,991,605	21,866,954	257,697 (0.59%)
FANG2_uncleaned_polyA	F2_PA	TAGCTT	61,297,778	60,912,867	30,328,346	256,175 (0.42%)
FANG3_uncleanedRZ	F3_RZ	GCCAAT	55,329,478	55,003,414	27,346,953	309,508 (0.56%)
FANG3_uncleaned_polyA	F3_PA	GGCTAC	60,735,310	60,465,080	30,105,533	254,014 (0.42%)

[1] Forward (R1) and reverse (R2) read pairs after trimming.
[2] Reads unpaired after trimming (% of total trimmed reads).

Figure 4. Trimmed read length distribution for forward (R1) and reverse (R2) reads and reads left unpaired after trimming (R0). A sample of approximately 1% of R0 reads and 0.1% of R1 and R2 reads was used in each case. Grey boxes represent the central 50% of read lengths and whiskers the extreme values. The maximum read length was 125 bp (i.e. no trimming).

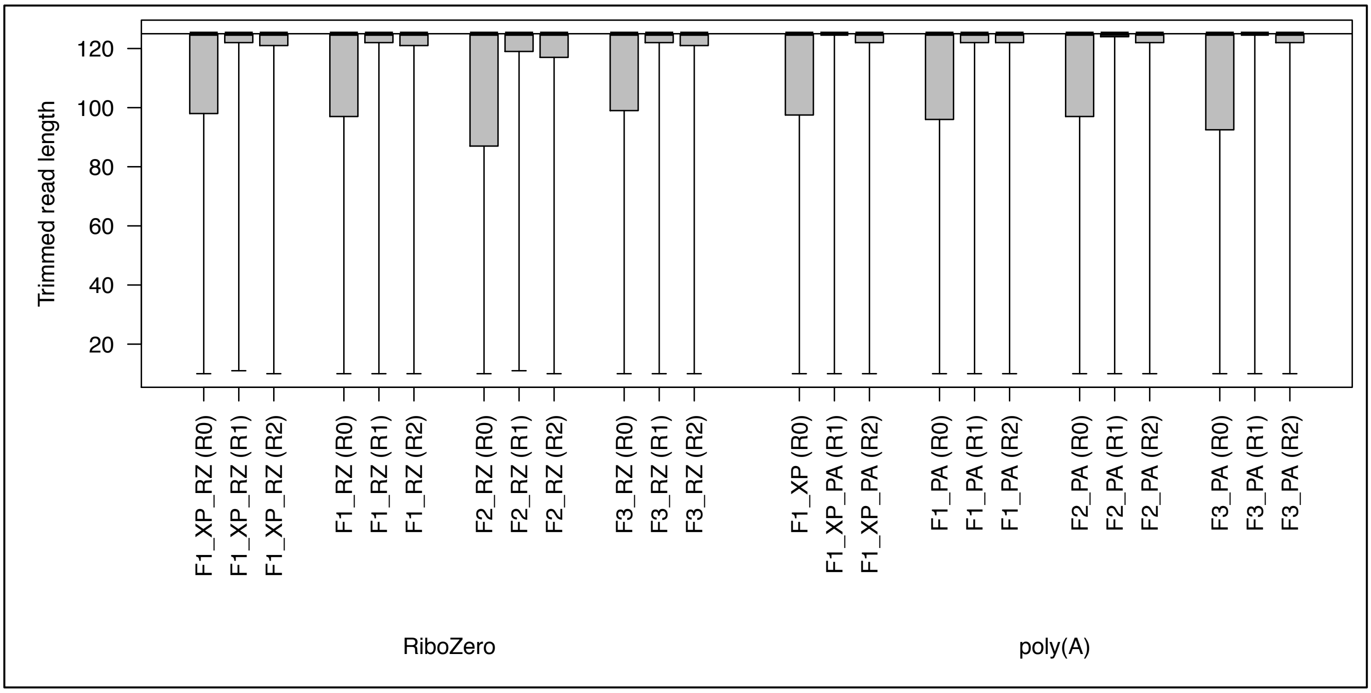

Analysis of gene expression data quality and reproducibility

Sequence reads were aligned to the reference genome and assigned to annotated genes as described in the Methods section of the main text. To assess the quality and reproducibility of the data for estimating gene expression levels, a number of analyses were carried out. Pairwise scatterplots of raw tag counts (a 'tag' being a read pair or single read representing a single fragment of RNA) per gene (not normalised for total library size) aligned in the sense orientation are shown in Figure 5. These plots show that sample clean-up prior to mRNA enrichment and library preparation had little effect on the results (e.g. "F1_XP_RZ" *vs.* "F1_RZ"), but the mRNA enrichment method had a greater effect (e.g. "F1_XP_RZ" *vs.* "F1_XP_PA"). The correlation coefficients of each scatterplot are represented as a heatmap in Figure 6. This shows that the poly(A)-enriched replicates correlate most closely with each other, followed by the Ribo-Zero replicates. The lower correlation among the Ribo-Zero samples is largely due to FANG-2 which correlates more poorly with the other two samples than they do with each other. The lowest correlation is seen between samples enriched using the two different methods. Principal component analysis was applied to the tag counts to show the relationships among samples. Loading plots are shown in Figure 7. The first principal component of variation (PC1) dominates the total variation (accounting for 96% of total variation). It clearly separated poly(A) from Ribo-Zero samples, possibly due to the larger total tag counts in poly(A) libraries (samples are not normalised for total library size, so we would expect this to dominate PC1). PC2 also clearly separates poly(A) from Ribo-Zero samples. PC3 separates FANG-2 (Ribo-Zero) from the other Ribo-Zero samples, reflecting the results seen in Figures 5 and 6. Overall, the data are of good quality and reproducibility (except for the aberrant FANG-2 Ribo-Zero sample). The data all indicate large differences between the transcriptome generated from poly(A) or from Ribo-Zero mRNA-enriched samples.

Differential gene expression analysis was carried out as described in the Methods section of the main text.

Figure 5. Scatterplots of log_2 sense tag counts per gene for 8 transcriptome samples. In the matrix of plots, sample data plotted on the x and y axes are indicated on the diagonal line. (Plots above and below this line are mirror images). Each data point represents an annotated gene.

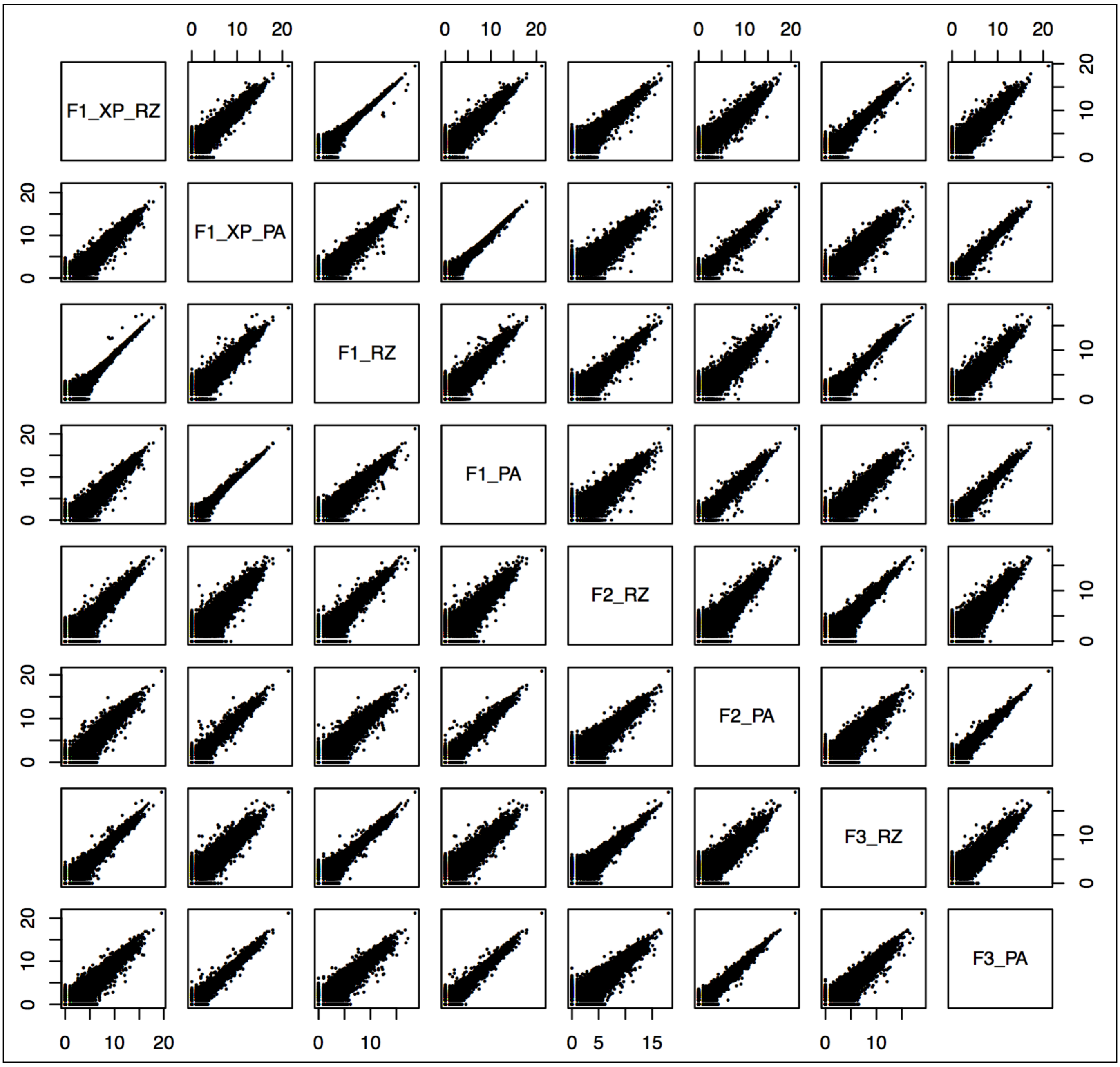

Figure 6. Correlation heatmap of Pearson's correlation coefficients among $\log_2$ sense tag counts. Samples correlate by mRNA enrichment method rather than by biological. Ribo-Zero samples correlate more poorly with each other (r^2 of 94.8% to 96.8%) than do poly(A) samples (r^2 of 98.2% to 98.8%).

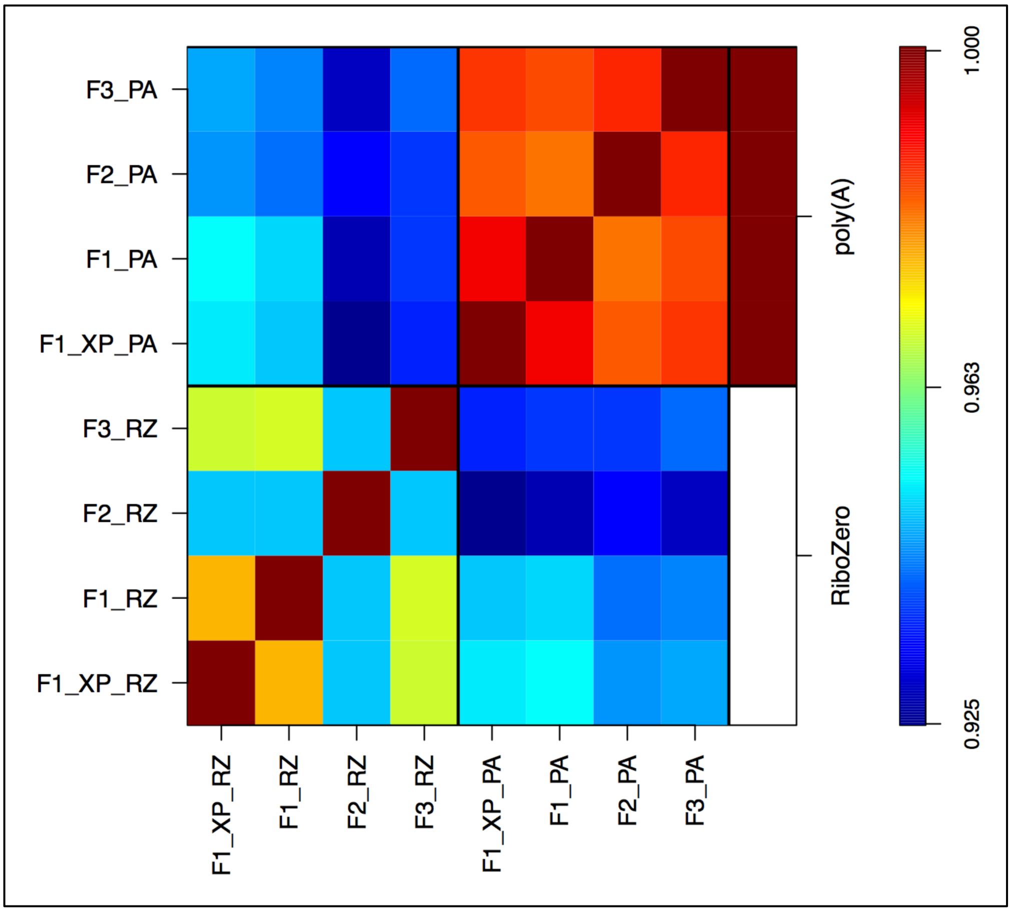

Figure 7. PCA loading plots of log_2 sense tag count data for the first (96.10% of the total variation), second (1.95%) and third (0.77%) principal components of variation in the data. (A) first and second principal components; (B) second and third principal components; (C) first and third principal components.

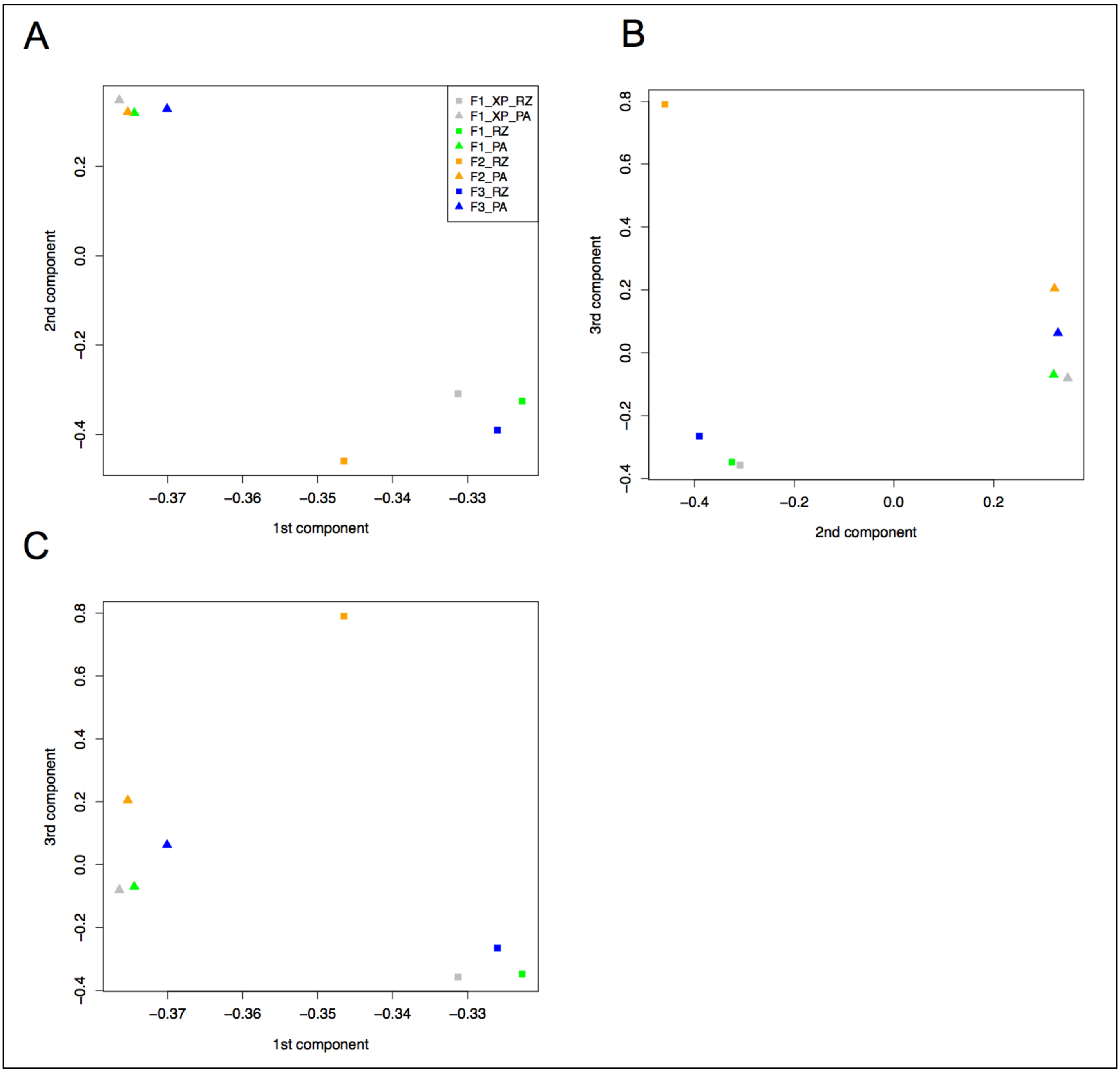

Analysis of differential transcriptome composition between mRNA enrichment methods

Transcriptome composition differences between poly(A) and Ribo-Zero mRNA-enriched samples were analysed using broad functional categories including gene ontologies (GO), as described in the main text. GO terms significantly differentially represented between gene sets significantly differentially represented in poly(A) and Ribo-Zero samples were shown in barplots of the proportion of the gene set associated with a given GO term. Figures 8 and 9 show these barplots for the 'biological process' and 'molecular function' GO domains. Results for the 'cellular compartment' domain are shown in the main text. All plots show over-representation of ribosomal proteins (also associated with 'translation' and 'structural constituent of ribosome' GO terms) in the poly(A) samples and of membrane-associated and/or nuclear proteins such as transporters and signal transduction-associated proteins in the Ribo-Zero samples.

Figure 8. Biological process GO terms significantly differentially represented between gene sets over-represented in poly(A) (red) and Ribo-Zero (blue) samples.

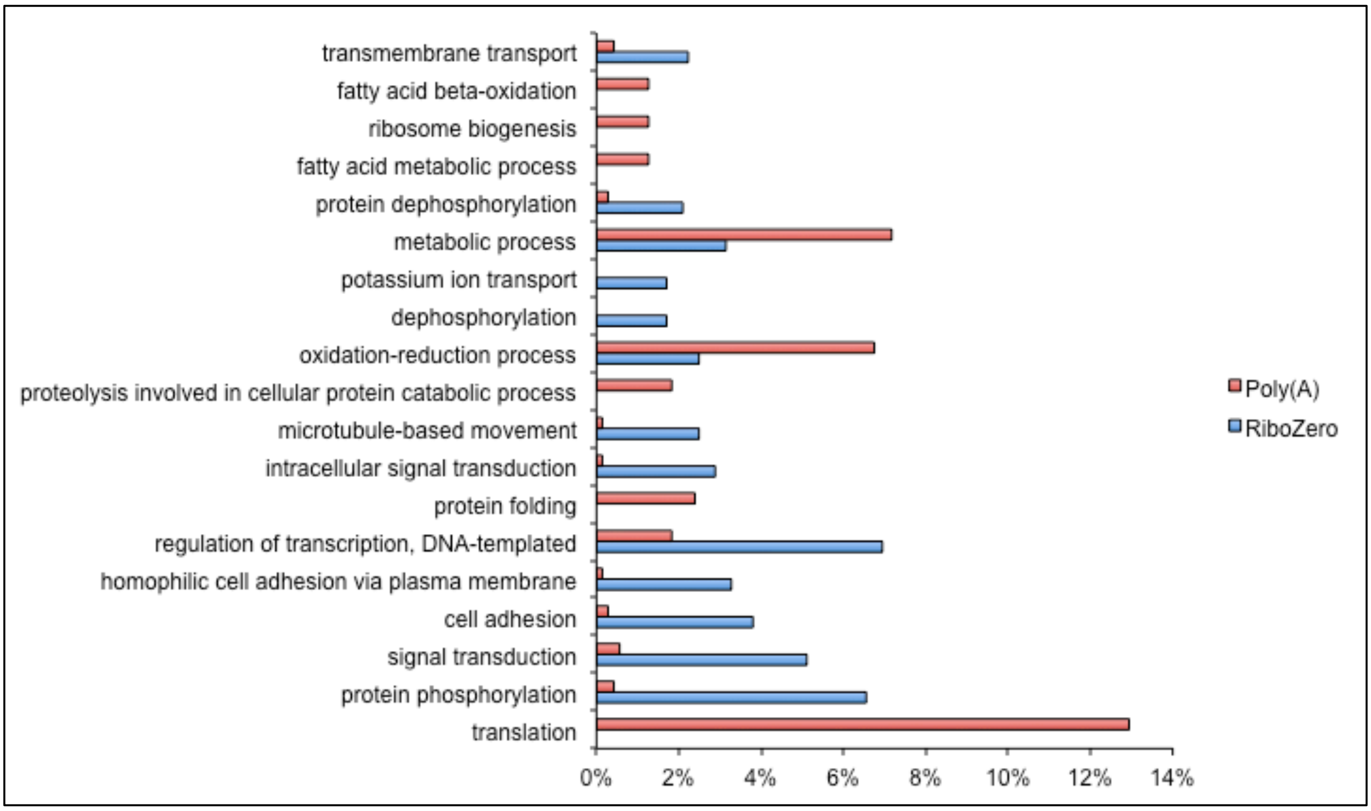

Figure 9. Molecular function GO terms differentially represented between gene sets over-represented in poly(A) (red) and Ribo-Zero (blue) samples.

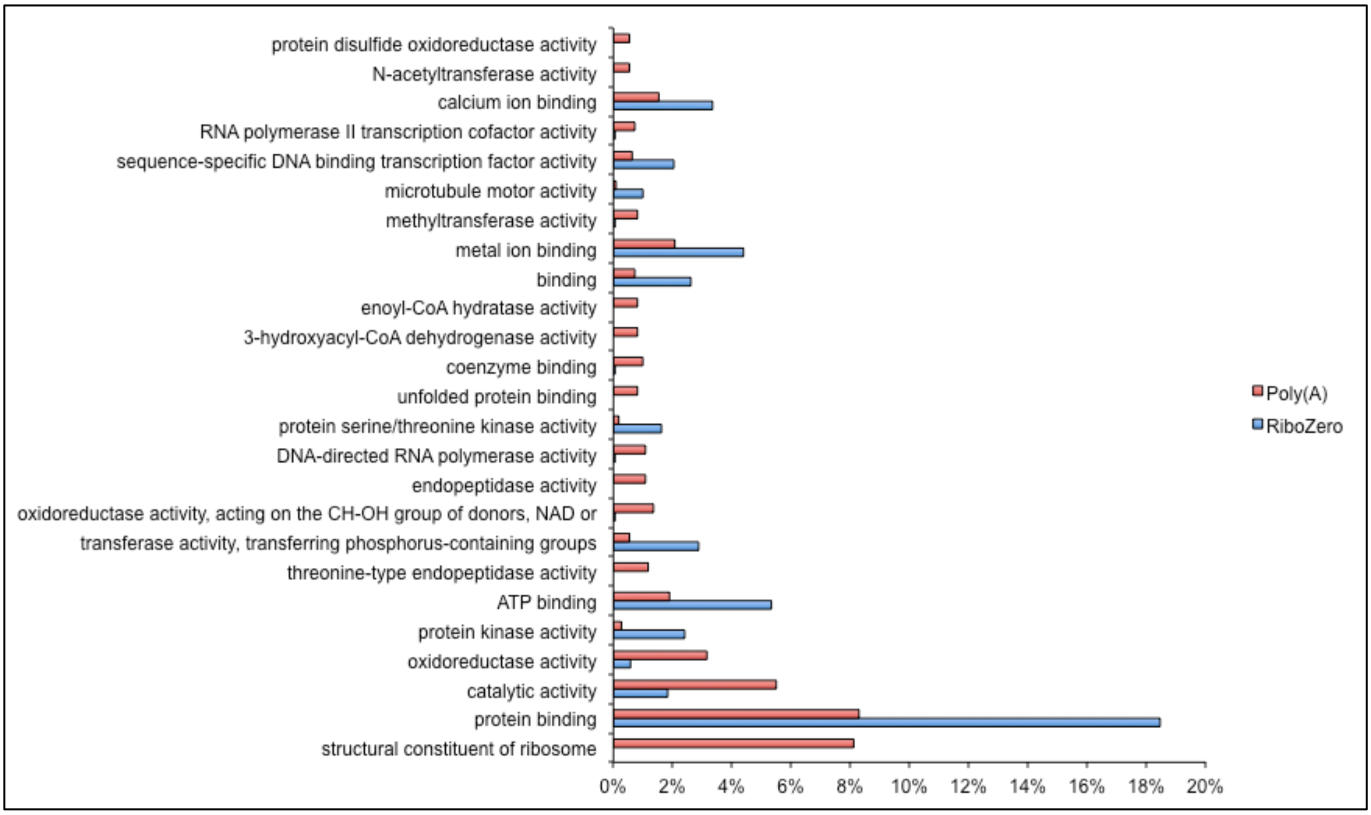

Supplement: Additional file: 1: — Detailed and extra information and figures. File containing detailed descriptions of the analyses and figures not included in the main article. (PDF 3540 kb) [file 12864_2015_2114_MOESM1_ESM.pdf]
